# Novel MRI Contrast Agent from Magnetotactic Bacteria Enables *In Vivo* Tracking of iPSC-derived Cardiomyocytes

**DOI:** 10.1038/srep26960

**Published:** 2016-06-06

**Authors:** Morteza Mahmoudi, Atsushi Tachibana, Andrew B. Goldstone, Y. Joseph Woo, Papia Chakraborty, Kayla R. Lee, Chandler S. Foote, Stephanie Piecewicz, Joyce C. Barrozo, Abdul Wakeel, Bradley W. Rice, Caleb B. Bell III, Phillip C. Yang

**Affiliations:** 1 Division of Cardiovascular Medicine, Stanford University, Stanford, CA, USA; 2 Nanotechnology Research Center, Faculty of Pharmacy, Tehran University of Medical Sciences, Tehran, Iran; 3 Department of Cardiothoracic Surgery, Stanford University, Stanford, CA, USA; 4 Bell Biosystems Inc., San Francisco, CA 94107, USA.

## Abstract

Therapeutic delivery of human induced pluripotent stem cell (iPSC)-derived cardiomyocytes (iCMs) represents a novel clinical approach to regenerate the injured myocardium. However, methods for robust and accurate *in vivo* monitoring of the iCMs are still lacking. Although superparamagnetic iron oxide nanoparticles (SPIOs) are recognized as a promising tool for *in vivo* tracking of stem cells using magnetic resonance imaging (MRI), their signal persists in the heart even weeks after the disappearance of the injected cells. This limitation highlights the inability of SPIOs to distinguish stem cell viability. In order to overcome this shortcoming, we demonstrate the use of a living contrast agent, magneto-endosymbionts (MEs) derived from magnetotactic bacteria for the labeling of iCMs. The ME-labeled iCMs were injected into the infarcted area of murine heart and probed by MRI and bioluminescence imaging (BLI). Our findings demonstrate that the MEs are robust and effective biological contrast agents to track iCMs in an *in vivo* murine model. We show that the MEs clear within one week of cell death whereas the SPIOs remain over 2 weeks after cell death. These findings will accelerate the clinical translation of *in vivo* MRI monitoring of transplanted stem cell at high spatial resolution and sensitivity.

Cardiac ischemia initiates a cascade of irreversible cell damage leading to cell death, regional contractile dysfunction, and progressive replacement by scar tissue. During this disease process, cardiac progenitor cells (CPCs) have been reported to migrate to the injured site, differentiate into cardiomyocytes, and eventually regenerate the myocardium^1^,^2^,^3^,^4^. However, the native population of CPCs is extremely limited and decreases significantly during the aging process^5^, compromising the myocardial repair potential. In order to compensate for the lack of CPCs in the injured site, therapeutic delivery of autologous patient-specific human induced pluripotent stem cell (iPSC)-derived cardiomyocytes (iCMs) has been proposed, generating promising outcomes in pre-clinical studies^6^,^7^,^8^,^9^,^10^,^11^.

Although intense efforts are made to track the fate of the transplanted stem cells, the inability to visualize the precise spatial location and viability of the delivered cells in the heart is recognized as one of the major limitations for clinical translation of cell therapy^12^,^13^. Such information is vital to determining the engraftment of iCMs and assess their therapeutic efficacy *in vivo*. Thus, it is essential to develop the novel imaging techniques to conduct translational investigation of the iCMs^14^,^15^. An ideal, non-invasive imaging platform should demonstrate high sensitivity, strong tissue contrast, and high spatial and temporal resolution. It is strongly preferred for imaging agents to be retained in the cells for longitudinal visualization of live cell specificity to confirm the engraftment of the delivered cells within the host myocardium. Optical reporters, requiring fluorescent or bioluminescent protein expression, are commonly used to label cells and can be effective in pre-clinical models. However, these methods are restricted by limited depth penetration and genetic modification of cells, preventing clinical translation. MR imaging has demonstrated exquisite capability to detect labeled cells in the myocardium without the ionizing radiation associated with positron emission tomography (PET) and single-photon emission computed tomography (SPECT) techniques^15^,^16^,^17^,^18^,^19^,^20^. The most commonly used MRI contrast agents for tracking transplanted stem cells, *in vivo*, are superparamagnetic iron oxide nanoparticles (SPIO). The limitation of SPIO cell labeling is the persistence of *in vivo* signal even after death of the transplanted cells where the MRI signal does not correlate with the viability of the stem cells^21^. For example, Ferumoxide (commercial SPIO) labeled stem cells were shown to persist in the heart muscle weeks after disappearance of concomitant beta-galactosidase or BLI signal, indicating labeling of dead cells or macrophages at the site of inflammation^21^,^22^. Another consideration is the dilution of the SPIO signal as the stem cells divide caused by nanoparticles being re-distributed among the dividing cells, making it difficult to track the biology of delivered cells in longitudinal studies^23^.

In order to overcome these issues, we evaluated the novel magneto-endosymbiont (ME) contrast agent (Magnelle® reagents Bell Biosystems, Inc., CA), which was derived from the magnetotactic bacteria *Magnetospirillum magneticum* strain AMB-1 (AMB-1). AMB-1 coordinates over 100 genes to synthesize membrane enclosed magnetic nanoparticles, (magnetosomes), which are highly effective MRI contrast agents^24^. In metastatic breast cancer cells, ME-labeled cells show strong contrast with as few at 100 ME-labeled cells, demonstrating high sensitivity for detection^25^. More importantly, the MEs do not appear to persist long-term in macrophages, eliminating any confounding MRI signal of cell viability. MEs are digested in the macrophages and cleared from the tissue, leading to rapid removal of non-specific dephasing MRI contrast. Thus, the MEs will persist only in the viable cells to provide accurate longitudinal engraftment signal of the transplanted stem cells.

## Results

### Cell Labeling with Contrast Agents

**Figure 1:**
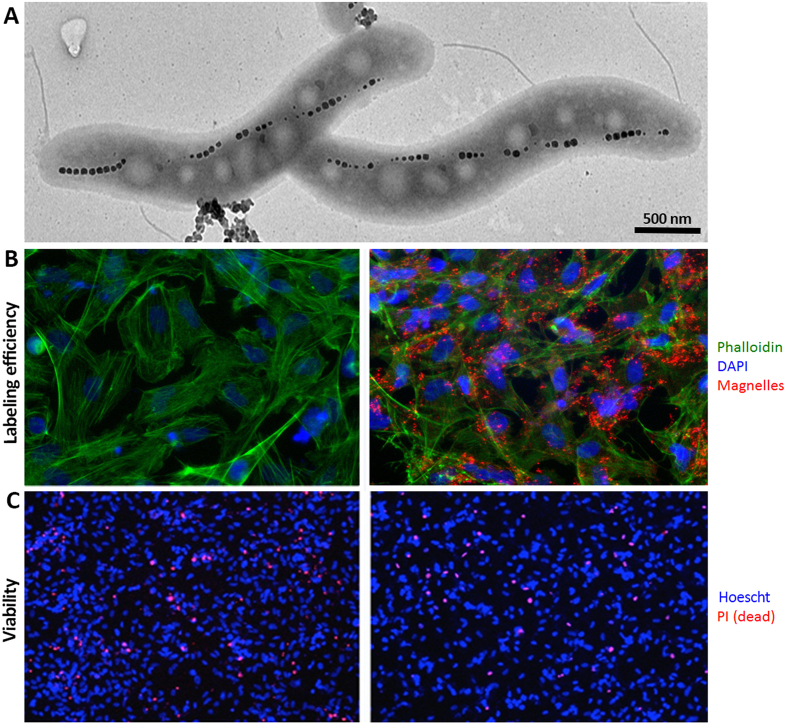
(**A**) TEM image showing the magnetosome structure within MEs; (**B**) fluorescent images of the unlabeled (left panel) and ME labeled (right panel) iCMs stained with ME antibody in red, phalloidin in green and DAPI in blue, showing internalization of MEs in the iCMs; and (**C**) viability assessment of unlabeled (left panel) and ME labeled (right panel) iCMs using PI assay, dead cells are shown with a red signal while blue Hoescht staining corresponds to the total cell population.

The morphology of the magnetic nanoparticle (magnetosome) chains inside the Magnelle was evaluated by transmission electron microscopy (TEM) as shown in Fig. 1A. Using the TEM images, the magnetosomes were measured and found to have an average diameter of 53 nm with about 31 magnetosomes per chain within a single Magnelle. Assuming the magnetosomes are 100% magnetite, we calculate 11.75 fg of iron per Magnelle using this particle counting method. Inductively coupled emission spectroscopy (ICP) measurements confirmed similar iron content per ME.

**Figure 2:**
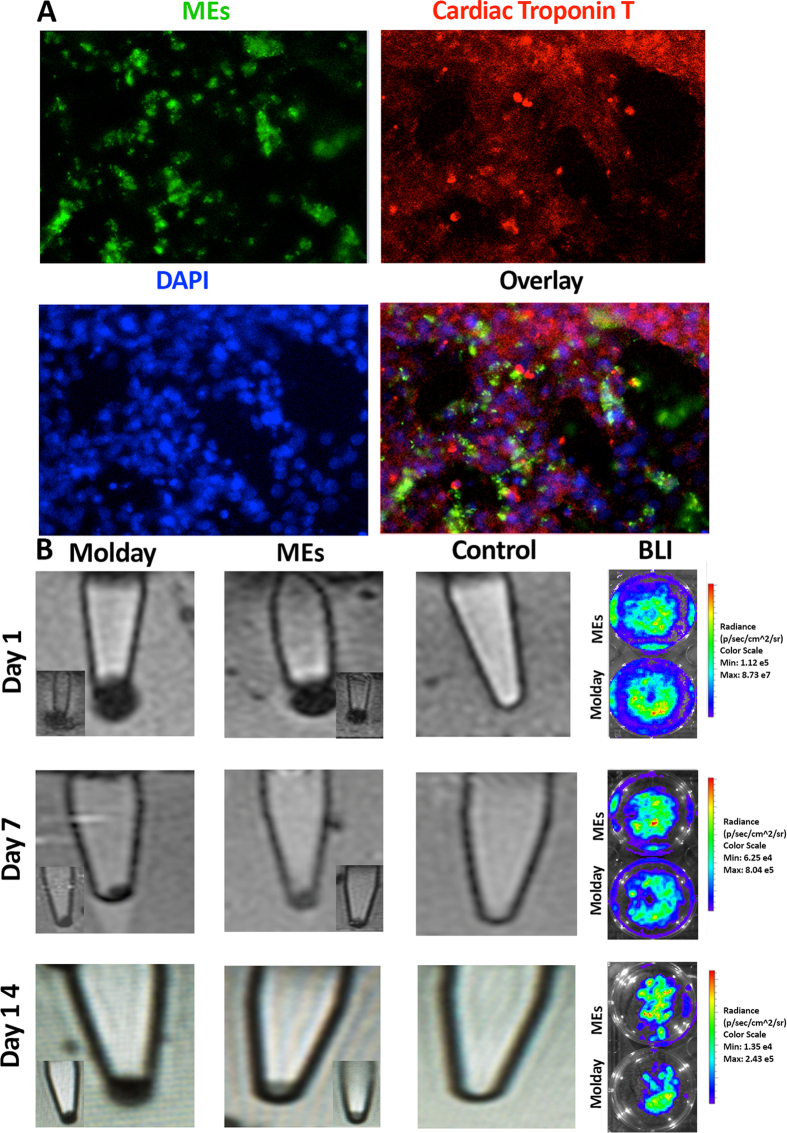
(**A**) Fluorescent images of the ME-labeled iCMs used for the evaluation of potential ME effects on cardiac marker, ME staining in green, cardiac troponin T in red, and nuclei are counterstained with DAPI in blue and (**B**) MRI images (T_2_^*^) of the bottom of Eppendorf tubes of Molday ION labeled iCMs, ME-labeled iCMs, and PBS only control sample at different time points (days 1, 7, and 14 after labeling); the bottom panel shows the replicate MRI images for Molday ION and ME labeled cells. Right panel shows the corresponding BLI signal, indicating viability of the Molday ION and ME-labeled iCMs at days 1, 7, and 14 post labeling (the BLI experiments were performed in triplicate).

MEs were then added at different concentrations to label iCMs. Immunocytochemistry, using a ME specific antibody to measure the ME concentration, showed the labeling efficiencies of the iCMs as demonstrated in Fig. 1B. For this condition, ICP measurements showed an average of 5.07 pg of Fe per cell, which was equivalent to about 400 MEs per cell. In order to evaluate potential adverse effects of MEs on the cell viability, we employed propidium iodide (PI) staining assay. As PI is a membrane impermeable dye, it cannot enter viable cells with intact membranes. In the case where iCMs membranes are perturbed due to the death of the cell, the PI is integrated in the DNA resulting in a red signal. As demonstrated by the PI staining in Fig. 1C, there was no significant difference between control unlabeled cells, which had a viability of 88% ± 2 and ME labeled cells which had 93% ± 3 cell viability (p > 0.05), showing no decrease in viability of cells after labeling with MEs. Additionally, the ME labeled cells maintained spontaneous contractility and exhibited positive staining for sarcomeric protein (i.e., cardiac troponin T). This finding demonstrated that the MEs do not affect the short-term cardiac properties of the cells after labeling (Fig. 2A).

### *In vitro* BLI and MRI evaluation of the labeled cells

In order to assess the contrast of the iCMs labeled with MEs, we performed *in vitro* MRI on days 1, 7, and 14 and compared this signal to the Molday ION labeled iCMs. The phantom experiment in Fig. 2B showed strong T2^*^ signal of the labeled cells (with both Molday IONs and MEs) on days 7 and 14 after labeling as seen by the dark pellets. Viability of the cells at different time points was monitored by BLI. The bioluminescent signals, exhibited in the right panels of Fig. 2B, demonstrated existence of healthy cells in the plates after labeling with both Molday ION and MEs, even after prolonged exposure.

### Evaluations of ME toxicity in animal model

**Figure 3:**
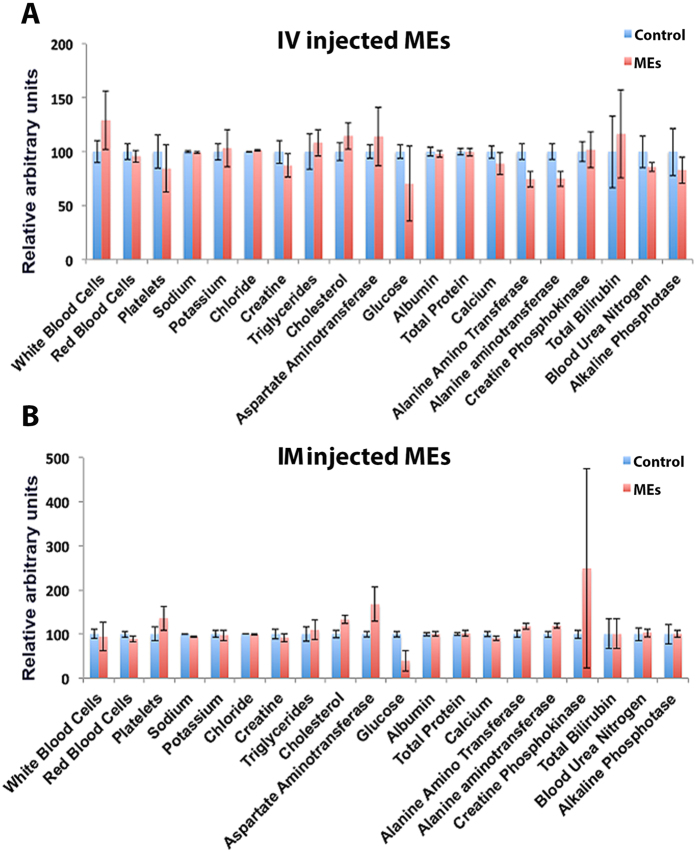
Relative concentrations liver and spleen enzymes and as well as key indices of infection and toxicity in blood did not changes in the highest doses of MEs (5.0E + 09) injected by both IV (**A**) and IM (**B**) routes.

Overall no external clinical signs were observed post-ME injection. Serum analysis of multiple markers assessing malfunction of vital organs such as the liver, kidney, spleen, heart and pancreas were performed for each group. The markers evaluated were alanine aminotransferase (ALT), creatinine, creatine phosphokinase, albumin, total protein, glucose, alkaline phosphatase (ALP), aminotransferase (AST), cholesterol, potassium, sodium, triglycerides, blood urea nitrogen (BUN), total bilirubin, calcium, total protein and chloride (see Fig. 3). All blood markers seemed normal indicating no malfunction of vital organs. Also, key markers of infection such as the white blood cells, red blood cells and platelet count did not seem to be altered in ME injected rats, suggesting that MEs probably do not trigger an immune response *in vivo.*

Pathological evaluation of tissue samples did not show significant differences in histopathology scores for the brain, kidney and heart. However, the intravenous (IV) injected rats showed signs of liver inflammation and both the intramuscular (IM) and IV injected groups showed significant spleen lymphoid hyperplasia (see supplementary Fig. S1). These symptoms are common during particulate material clearance by these tissues and the dose used in this study was 10-fold higher that the working dose of MEs. Together, the data from these studies demonstrated that MEs were most likely not immunogenic and, therefore, feasible for *in vivo* studies.

### *In vivo* BLI and MRI evaluation of labeled cells

**Figure 4:**
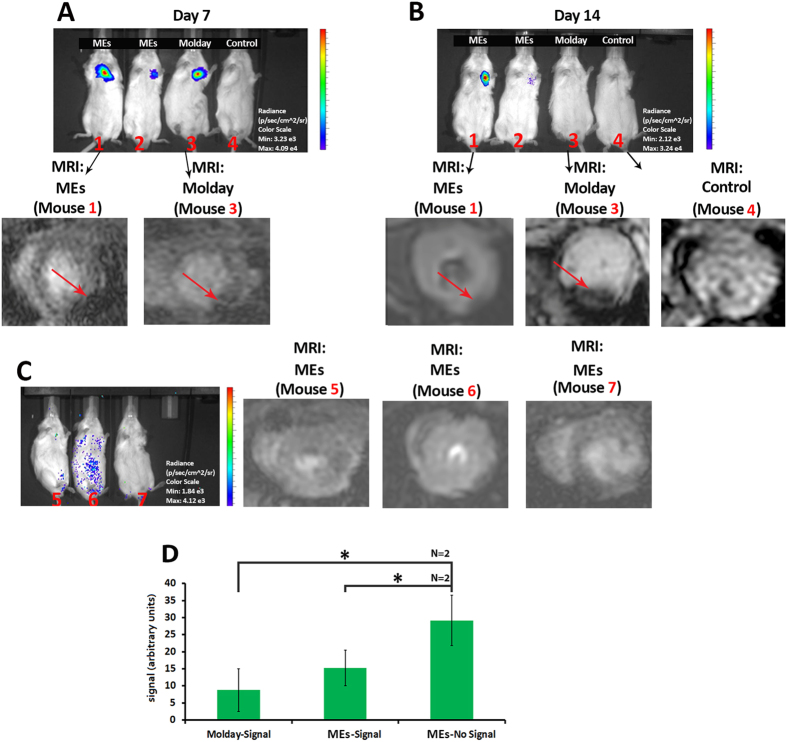
Representative BLI images of the mice injected with ME and Molday ION labeled reporter gene-transduced iCMs or PBS control after (**A)** 7 days; (**B**) 14 days following cell injections (top) and their corresponding *in vivo* MRI images of the murine hearts (bottom). Red arrows show the signal from the injected labeled cells; (**C**) representative *in vivo* BLI images of the selected mice with dead cardiomyocytes at day 14 of ME labeled cells and the corresponding *in vivo* MRI images; (**D**) signal intensity (SI) for Molday ION (dark signal) and ME (with no signal). A significant absence of signal of ME* labeled dead cells compared to other samples with positive signal (p < 0.05).

After the injection of labeled iCMs into a live mouse heart as shown in Fig. 4, both bioluminescence and MRI signal from the injected cells could be easily detected seven days after cell injection. Over time a diminished BLI signal is observed in all mice models, mainly due to cell death and removal by the immune system^26^. Mice #2 and #3 show BLI signal clearance after a week while mouse #1 retains BLI signal through 2 wks. This variability in stem cell survival is typically seen when using a murine model of myocardial injury.

Importantly, the Molday ION labeled cells indicated no BLI signal by day 14; however, the cells continued to show dephasing MRI signal consistent with the persistence of the nanoparticle signal in the murine heart. This finding demonstrated the lack of live cell specificity with the inert, non-biological properties of Molday ION. In contrast to the Molday ION labeled cells, the ME labeled iCMs demonstrated significant correlation between BLI and MRI signals *in vivo*. In the mice with BLI signal at day 14 (mouse #1) post-cell injection, we could detect their corresponding MRI signals in Fig. 4B. However, in those mice with no BLI signal, there was no/negligible corresponding dephasing signal in the MRI scans as well as shown in Fig. 4C. The results were further confirmed by the evaluation of the cellular signal intensity (SI) as shown in Fig. 4D. While further validation is necessary, the correlation of the *in vivo* MRI signal with the engrafted iCMs may enable longitudinal evaluation of the survival and engraftment kinetics of the stem cells at exquisite spatial and temporal resolution with high sensitivity and tissue contrast.

## Discussion

Innovative technologies to evaluate stem cell engraftment is required for clinical translation of cell therapies. Evaluation of stem cell engraftment in the myocardium characterizes the most fundamental process of post-transplantation stem cell biology. With the potential emergence of cell therapy for a failing heart, accurate *in vivo* evaluation of stem cell engraftment and the resultant changes in the myocardial viability may represent a critical measure of therapeutic efficacy. The stem cells at the very least must survive to restore the injured myocardium^27^,^28^,^29^,^30^. Therefore, cell engraftment monitoring holds physiologic relevance as it has been associated with myocardial restoration^31^,^32^,^33^. Reliable and precise cell tracking technique represents one of the key issues in advancing the field of cardiovascular cell therapy. However, there are no reliable *in vivo* imaging methods to confirm definitively that this fundamental post-transplantation biology contributes to cardiac function due to two technical considerations: (1) sensitive detection of the cell engraftment signal and (2) high spatial and temporal resolution imaging of the myocardial function and morphology. MRI combines the chemical sensitivity of nuclear magnetic resonance with high spatial and temporal resolution. It routinely offers sub-millimeter spatial resolution, millisecond range temporal resolution, and intrinsically superior contrast mechanism. These specifications provide optimal technical characteristics to assess myocardial physiology. However, MRI lacks the sensitivity to detect the transplanted cells. Despite the high sensitivity provided by the magnetic nanoparticles for cell tracking applications, *in vivo* monitoring of the cell viability in the heart tissue has not been possible due to the confounding signal of the nanoparticles in the tissue or the macrophages after the death of transplanted stem cells^34^,^35^. In order to address this major limitation, we employed a novel MRI contrast agent, MEs, an endosymbiotic bacterium which synthesizes magnetosomes.

Our cytotoxicity evaluation demonstrated no sign of cell toxicity in the ME labeled iCMs. Furthermore, the toxicity studies in the rat models, which underwent ME injection, showed minimal effects in the relevant dose levels. The signal generated from the *in vitro* and *in vivo* BLI and MRI studies showed corresponding evidence of robust contrast to track the iCMs. Seven days following cell injections, both ME and Molday ION labeled cells showed significant MRI and corresponding BLI signal in the murine myocardium (Fig. 4A,B). By day 14, the Molday ION labeled cells did not express BLI signal demonstrating cell death. Yet, these mice exhibited strong MRI signal, demonstrating the major limitation of synthetic iron oxide nanoparticles for precise *in vivo* monitoring of live cells in the heart muscle as demonstrated in our and other publications^21^,^22^,^30^. However, in the ME mice with no iCM survival as shown by the absence of BLI signals, no/negligible MRI signal was detected, confirming the rapid removal of magnetosomes after cell death (Fig. 4C,D). The main reason for the fast removal of the ME signal, after the death of the labeled cells, may be attributed to their interactions with immune system. It has been shown that the magnetic particles (obtained from magnetotactic bacteria) were eliminated from the circulation by the immune system following their administration into the vascular system of rat model^36^,^37^. Based on previous studies by Sun *et al*.^36^, and Liu *et al*.^27^, this live cell sensitivity (LCS) emerged through a macrophage mediated mechanism. The studies with MTB and purified magnetosomes suggested that both lysosomal digestion and clearance mediated by the reticuloendothelial system targeted the magnetosomes into the free iron pool or eliminate them into the feces. The reticuloendothelial system mediated ME clearance may be significantly expedited by the antigenic nature of the MEs in comparison to the IONs, which have an inert coating and precipitate in the tissue post cell death. Histological examination of the major organs showed the existence of a large portion of magnetotactic bacteria derived nanoparticles in liver with increased numbers of vacuoles^37^. Researchers revealed that the existence of biological impurities (particularly proteins, nuclei acids, and polysaccharides), which was exposed after the death of the stem cells, led to rapid extraction of the magnetotactic bacteria and their associated magnetic particles by the immune system^36^,^37^.

Our study showed for the first time the use of MEs, to track the iCMs *in vivo* by MRI. Recent advances in regenerative medicine demonstrated the vast potential of cell therapies for a wide range of therapeutic areas such as neurodegenerative disease, myocardial infarction, oncology, immunology and spinal cord injury. However, the methods to track these cells after delivery had been a limiting factor for rapid clinical translation. Here we showed a novel contrast agent for MRI-based cell tracking that allows longitudinal visualization of the cells in a murine model. Most importantly, the use of the ME suggested LCS, which may allow significant advantage over commercially available IONs such as the Molday IONs as shown here. This novel technology may allow researchers to effectively track live cells *in vivo* to monitor their engraftment and distribution, which is critical to assess their functionality and therapeutic effect. It is noteworthy that the experiments should be applied in larger animals such as a porcine model to study the feasibility of clinical translation. Specific issues, including the sensitivity of MRI and dosing limitation of ME, should be investigated to optimize the dose for reliable and precise signal for cell tracking applications. In conclusion, this study demonstrated an innovative and necessary stem cell tracking technology to advance the field of regenerative medicine.

## Materials and Methods

### Preparation of iCMs from RG-transduced iPSCs

Zinc finger nuclease (ZFN) technology (Sangamo, Inc., Richmond, CA) was used to integrate a double fusion reporter gene, containing firefly luciferase (BLI) and herpes simplex virus thymidine kinase, under the regulation of a cardiac-specific CCAG promoter, into the AAVS1 locus (chromosome 19) of iPSCs^38^. The iPSC monoclonal lines were then differentiated into iCMs. Differentiation into iCMs was performed under chemically defined conditions using small molecules, CHIR (4 μM for 2 days) and C59 (2 μM for 2 days), to modulate Wnt pathway activity^6^,^39^. The cells were then treated with basal media (RPMI + B27) for another 6 days to reach the beating state. The purification of iCMs were performed by treating the cells with low glucose media (as previously described)^39^.

### Cell Labeling with MEs and Molday ION

MEs (Magnelle® cell tracking solution, Bell Biosystems, San Francisco, CA, USA) and Molday ION Rhodamine B (Biopal Inc, Worceshter, MA, USA) were used to label iCMs. Molday ION was used at a concentration of 25ug[Fe]/ml and MEs were added at a ratio of 2500 MEs per iCM cell. The cells were labeled by overnight incubation in a MagTag-6 system (Bell Biosystems, San Francisco, CA, USA) at 37° C with 5% CO_2_ and 5% O_2_. The MagTag-6 system utilizes an array of 6 strong permanent magnets to pull the MEs against the host cell surface, thus improving uptake. The following day the cells were washed and treated with a 1:1000 dilution gentamycin sulfate, 50 mg/ml (EMD Millipore) to kill extracellular MEs (additional information described elsewhere)^40^.

### Immunofluorescence microscopy

Magnelle labeled iCMs were fixed with 4% paraformaldyhyde and permeabilized with 0.25% tritonX. Samples were stained with a rabbit polyclonal anti-Magnelle primary antibody (Bell Biosystems, San Francisco, CA, USA) and an Alexa Fluor 488 or 594 –conjugated donkey anti-rabbit IgG (Invitrogen) secondary antibody. Alexa Fluor 488 Phalloidin was used to stain actin and VECTASHIELD mounting media with DAPI was used to mark for nucleic acid staining (additional information described elsewhere)^40^. The cardiac marker troponin T (ThermoFisher) primary antibody was used for iCMs to assess cell fate post labeling. Cell viability was assessed with a Propidium Iodide (PI) Solution (Biolegend) to label dead cells and a Hoescht 333482 counterstain was used nuclei and give the total cell population. Percent viability was calculated by dividing the live cell count (total minus the PI positive count) by the total number of cells. All immunofluorescence was imaged using a Nikon ECLIPSE Ti-s (Tokyo, Japan) microscope.

### Inductively coupled emission spectroscopy (ICP) Measurements

Following labeling, TrypLE was used to dislodge cells from the plate. Cells were washed with PBS by centrifugation and then counted. For ICP measurments, cells or MEs alone were pelleted and resuspended in 65% nitric acid and heated at 95° C for 20 min. Once cooled the volume was brought up to 2 mL with sterile DI water. Samples were sent to the Center for Applied Isotope Studies at the University of Georgia for intracellular iron quantification.

### Evaluation of ME toxicity in animal model

Although MEs are derived from non-pathogenic bacteria, there were concerns regarding their toxicity when released from the cells after injection into an animal. To address this issue, acute toxicity studies were performed in immune competent rats. ME reagents alone were injected at varying doses (low, medium and high with the highest does being two logs over the working dose) through various routes. For comparison, typical cell tracking in small animals would have 10^4^ to 10^8^ Magnelle reagents per animal as the number of Magnelle reagents per cell ranges from tens in small cells to thousands in very large cells. Therefore in this study the highest doses were: 1) Intravenous (IV) 5.0 × 10^9^ MEs in 500 μL, 2) Intramuscular (IM) 5.0 × 10^9^ MEs in 300 μL and 3) Intrathecal (IT) 5.0 × 10^8^ in 50uL. Rats (n = 5, per condition) were monitored for body weight and clinical signs were recorded thrice per week. At day 5-post injection, rats were euthanized and whole blood was collected via a terminal cardiac puncture for serum analysis. Also, tissue samples of vital organs such as the brain, heart, kidney, liver and spleen were fixed in formalin for pathological evaluation.

### Intramyocardial delivery of iCMs

Animal care and interventions were performed in accordance with the Laboratory Animal Welfare Act, and all animals received humane care and treatment in accordance with the “Guide for the Care and Use of Laboratory Animals” ( www.nap.edu/catalog/5140.html). All experimental protocols were approved by Administrative Panel on Laboratory Animal Care (APLAC) at Stanford School of Medicine. Immunotolerant SCID-beige male mice (6 weeks old; Charles River Laboratories, Inc, MA, USA) were anesthetized in an isofluorane inhalational chamber and endotracheally intubated with a 20-gauge angiocatheter (Becton, Dickinson and Co., NJ, USA). Ventilation was maintained with a MicroVent rodent ventilator (Hallowell EMC, MA, USA). A blinded surgeon injected 40 μl of dispersed labeled cells (500,000 cells) in PBS into the myocardium of the anterolateral wall of the heart via a left anterior thoracotomy.

### *In vivo* magnetic resonance imaging (MRI)

Cardiac MRI (Signa 3 T HDx, General Electric Medical Systems, WI, USA) was performed using a dedicated mouse coil (Rapid MR International, Germany). Mice were imaged at days 1, 7, and 14 after cell injection. Mice were anesthetized with 1-2% isoflurane at 2 L/min oxygen and placed in the supine position. Electrocardiographic gating was obtained with two subcutaneous precordial leads and body temperature was monitored with a rectal probe throughout the scan (SA Instruments, Inc, NY, USA). Left ventricular function was evaluated with electrocardiographically triggered fast spoiled gradient-recalled echo (FSPGR) sequence (TR 24 ms, TE 10 ms, FA 45, field of view (FOV) 6 cm^2^, matrix 256 × 256, slice gap 0 mm, slice thickness 1 mm, NEX 4, 2 excitations, and 20 cardiac phases). In order to define the relationship of signal intensity differences between the Molday ION and MEs regions, scaled to image noise, we measured the signals from Molday ION and MEs region (using OsiriX software) and divided them to the noise signal. It is noteworthy that the measurements were performed 5 times and the regions of interest’s size remained constant.

### *In vitro* magnetic resonance imaging

*In-vitro* cell scans were performed with gradient echo (GRE) sequence (TR 100 ms, TE 10 ms, FA 30, field of view (FOV) 6 cm^2^, matrix 192 × 160, slice gap 0 mm, slice thickness 1.3 mm, NEX 4).

### Optical Bioluminescence Imaging (BLI)

Optical BLI was performed 15-25 minutes after d-luciferin IP injection (400 mg/kg; PerkinElmer, MA, USA) with 3-5 minute acquisition scans on a charge-coupled device camera (IVIS 200; PerkinElmer). Peak signal from a fixed region of interest was evaluated with Living Image 3.2 software (PerkinElmer). For *in vitro* BLI, luciferin (16.6 mg/ml) was mixed with cell media in the tissue culture dish (10 minutes before imaging) and imaged.

## Additional Information

**How to cite this article**: Mahmoudi, M. *et al*. Novel MRI Contrast Agent from Magnetotactic Bacteria Enables *In Vivo* Tracking of iPSC-derived Cardiomyocytes. *Sci. Rep.* **6**, 26960; doi: 10.1038/srep26960 (2016).

## Supplementary Material

Supplementary Information
