# Supplementary material for: Novel MRI Contrast Agent from Magnetotactic Bacteria Enables In Vivo Tracking of iPSC-derived Cardiomyocytes
Source: Sci Rep. 2016 Jun 6;6:26960. doi: 10.1038/srep26960 (PMC4893600; doi:10.1038/srep26960)
Supplement: Supplementary Information [file srep26960-s1.doc]

**Supporting Information**

**Novel MRI Contrast Agent from Magnetotactic Bacteria Enables *In Vivo* Tracking of iPSC-derived Cardiomyocytes**

Morteza Mahmoudi^1,2^, Atsushi Tachibana^1^, Andrew B. Goldstone^3^, Y. Joseph Woo^3^, Papia Chakraborty^4^, Kayla R. Muth^4^, Chandler S. Foote^4^, Stephanie Piecewicz^4^, Joyce C. Barrozo^4^, Abdul Wakeel^4^, Bradley W. Rice^4^, Caleb B. Bell III^4^, Phillip C. Yang^1^*

^1^ Division of Cardiovascular Medicine, Stanford University, Stanford, CA, USA

^2^ Nanotechnology Research Center, Faculty of Pharmacy, Tehran University of Medical Sciences, Tehran, Iran.

^3^ Department of Cardiothoracic Surgery, Stanford University, Stanford, CA, USA

^4^ Bell Biosystems Inc., San Francisco, CA 94107

Address correspondence to:

Phillip C. Yang, MD

269 Campus Drive, CCSR 3115c

Stanford, CA 94305

Tel: 650-498-8008

Fax: 650-724-4034

[phillip@stanford.edu](mailto:phillip@stanford.edu)

Control ME(IV)


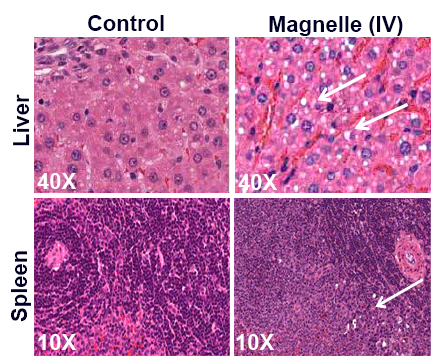


Figure S1: Hematoxylin and eosin stained tissue sections showing the effects of high doses (10^9) of Me injection by IV. Top panel shows sections of the mice liver. MEs injected sample (right) show liver inflammation and focal degeneration characterized by randomly scattered lymphocytes within lobules and small variously sized intracellular vacuoles (small white spaces- white arrow). Bottom panel shows spleen lymphoid hyperplasia in ME injected mice with some necrotic regions (white arrows). Liver images are presented at 40X magnification to aid visualization of the macrophages infilteration of tissues (red elongated cells) and spleen images are presented at 10X magnification to highlight the necrotic regions.
